# Supplementary material for: Mapping human visual contrast sensitivity and vision loss across the visual field with model-based fMRI
Source: eLife. 2026 Oct 2;14:RP105930. doi: 10.7554/eLife.105930 (PMC13633074; doi:10.7554/eLife.105930)
Supplement: Peer review history

# Editor report: eLife Assessment

**DOI:** 10.7554/eLife.105930.3.sa0

**Authors:** Xilin Zhang

**Affiliations:** Key Laboratory of Brain, Cognition and Education Sciences, Ministry of Education, South China Normal University China

**Keywords:** Convincing, Important

Using fMRI-based pRF mapping, this **important** study presents a novel method for estimating visual field (VF) loss and potential restoration by analyzing contrast-sensitivity patterns in early visual cortex. The evidence supporting the main claims is **convincing**. This work will be of broad interest to researchers in vision and clinical vision, neuroscience, and brain imaging.

# Referee report: Reviewer #1 (Public review)

**DOI:** 10.7554/eLife.105930.3.sa1

Integrating large-field stimulation with a retinotopic atlas, this study introduces an fMRI-based method for measuring contrast sensitivity across the visual field. Retinotopy was assessed using pRF mapping and a calibrated Benson atlas. The authors validate their method by replicating known patterns of contrast sensitivity across eccentricities and visual field quadrants in healthy subjects, and demonstrate its potential clinical utility through case studies of both simulated and real visual field loss.

Comments on revisions:

I appreciate the addition of the quadrant-scotoma condition and the authors' clarification that the goal is to demonstrate individual-level detection sensitivity. The 95% CI argument is reasonable, and I am satisfied with framing the simulated-scotoma work as proof-of-concept.

# Referee report: Reviewer #2 (Public review)

**DOI:** 10.7554/eLife.105930.3.sa2

Summary

This study uses functional MRI to evaluate visual contrast sensitivity across the visual field at the level of the visual cortex, testing the method as a proof of principle in a small group of normally sighted individuals, modelling both normal vision and simulated vision loss, as well as a patient with independently verified vision loss. The results suggest a promising technique to measure vision objectively across the visual field and overcomes the requirement for careful fixation which is often challenging in those with low vision or sight loss.

Strengths

• Objective measure of central vision: The proposed method may provide a more comprehensive and objective assessment of residual visual function in individuals with sight loss. This may be particularly useful for those with central visual field loss without the requirement of stable fixation or subjective motor responses.

• More sensitive measure: The use of slope to calculate contrast sensitivity across a range of contrasts within the brain is clever and likely more sensitive than single threshold measurements or standard clinical measures of visual acuity using letter charts. Standard supra-threshold (high contrast) tests are not ideal for capturing residual vision or partial vision loss.

• Good agreement with standard atlas: The Benson atlas provides a good estimate of visual field maps within V1 based on anatomical landmarks, and the authors take steps to refine this informed by cortical magnification and V1 surface area (brain size) for each individual participant. This could allow the technique to be generalised without the need to collect lengthy individual mapping data from every participant.

• Within-subject reproducibility: The measurements appear to be sensitive and reproducible, particularly in those with normal vision, and are consistent with known features of visual sensitivity differences in different parts of the visual field.

• Potential tool to measure visual field sensitivity in controls: Even if the proposed methods are not ideal for widespread clinical translation, they do offer an exciting tool to test hypotheses about visual field differences in healthy controls. For example, there seems to be an increase in sensitivity on either side of the simulated ring scotoma (Fig 6 - perhaps due to the release of lateral inhibition?). Reliability measures suggest that individual differences are consistent in healthy controls (although not tested statistically, perhaps due to the small sample size?). Whether they reflect behaviourally meaningful differences in visual field sensitivity could be tested in individuals by comparing them to behavioural measures across the visual field.

• Potential tool to test novel treatments: The proposed techniques could be used to test within-subject changes in visual function in environments that are equipped to measure and analyse fMRI data, including clinical trials aimed at determining the success of novel treatments. Preliminary testing in healthy controls with eye movements also suggests that the method is suitable for testing low vision patients with unstable fixation (e.g., nystagmus), and the authors have modelled the effects of varying amounts and types of eye movements on functional outcome measures.

Weaknesses

• Questionable sensitivity to differences in patients. The variability in heat maps across healthy control participants is somewhat surprising, and it is uncertain whether they represent actual visual sensitivity differences or an artifact of the measurement technique, e.g., due to signal-to-noise differences introduced by local variations in brain anatomy. Thus, it is uncertain whether the substantial variance across controls will allow for a sufficiently stable baseline to detect meaningful differences in individual patients. Also, as the authors rightly point out, Benson atlas does not model differences along meridians, so that upper/lower field differences might not be detectable. However, the authors acknowledge that this is a pilot study, and further testing a wider range of scotoma types in patients and simulated in controls will only improve the methods. Furthermore, the ability to capture visual field representations in human visual cortex is also likely to improve with computational advances, making the use of atlases more feasible, obviating the need for individualised population receptive field mapping.

• Potential for clinical translation. Although it is a sensitive measure, functional MRI is costly, is not available in all clinical settings, requires significant post-processing analyses, and may be contraindicated in some individuals due to safety (e.g., metallic implants) or other concerns (e.g., claustrophobia). These could present significant barriers to widespread clinical translation, if this were the ultimate goal of the study.

• Limited range of spatial frequencies. The spatial frequencies tested were still quite low (0.3 and 3cpd) compared to measures such a visual acuity. Extending the measurements to higher spatial frequencies could allow better characterization of central vision, although necessarily for peripheral vision. However, this may depend on the typical visual abilities of the patient population of interest.

Appraisal and Impact:

The authors used appropriate and robust methods to assess and model known features of visual sensitivity differences across the visual field in sighted controls. In addition, the assessment technique successfully captured sensitivity changes due to simulated and actual partial field loss but was also fairly resilient to eye movements and fixation instability, typical of patients with sight loss. Although currently providing a proof of principle, the method is likely to improve with further testing and increasing normative sample sizes, and as computational methods continue to advance visual field map predictions. Although it may not be adopted widely as a standard clinical assessment technique due to the expense and other obstacles, it would provide a valuable tool in assessing clinical populations, for example in the context of clinical trials to assess suitability for treatment interventions or monitor treatment outcomes.

# Referee report: Reviewer #3 (Public review)

**DOI:** 10.7554/eLife.105930.3.sa3

Summary:

Chow-Wing-Bom et al. introduce an innovative wide-field visual stimulation setup for 3T experiments that enables stimulation up to a diameter of 40{degree sign} visual angle while allowing continuous gaze tracking. Using this setup, the authors systematically investigate contrast sensitivity across the visual field by presenting subjects with sinusoidal gratings varying in contrast and spatial frequency. Their findings confirm the expected organization of contrast sensitivity, demonstrating a preference for high spatial frequencies in the central field and lower frequencies in the periphery. They also extend these measurements to eccentricities up to 20{degree sign}, which exceeds previous fMRI-based reports. Moreover, the study explores the potential of using contrast sensitivity calculations as a method for detecting visual field defects, demonstrated in a healthy subject with simulated ring-shaped and upper-right-quadrant scotomas, and in a patient with LHON. The revised version additionally characterises the robustness of the approach to varying degrees of fixation instability.

Strengths:

- The manuscript is well written and provides comprehensive methodological details, ensuring high transparency and reproducibility.

- The visual stimulation setup represents a significant technical advance by enabling wide-field stimulation with continuous eye tracking, which is crucial for both research and potential clinical applications.

- The study confirms established findings regarding the organization of contrast sensitivity while extending them to a larger eccentricity range.

- The efforts to establish a measure for visual field losses aligns with current efforts to develop objective alternatives to conventional perimetry.

- The revised manuscript includes an empirical assessment of how varying levels of eye movement affect cortical contrast sensitivity estimates, providing useful guidance on the tolerance of the approach to fixation instability.

Weaknesses:

- The original version left certain methodological aspects unclear, particularly the correction of eccentricity values from the Benson atlas and the V1 masks used in each analysis branch. The authors have added a dedicated figure illustrating the eccentricity correction procedure and now explicitly state that a manually delineated V1 mask was used for the pRF-based analyses while the Benson V1 label was used for the atlas-based analyses, together with a discussion of how this difference may influence the comparison.

- Minor inconsistencies in reporting, such as the introduction of a second session in the Results section, have been corrected.

The conclusion that high-contrast patterns as in pRF mapping are not optimal to test for subtle but potentially clinically relevant changes in the visual field coverage are very valid. The suggested use of contrast sensitivity can therefore be a potentially well-suited parameter for estimating visual field losses. The presented work is an interesting starting point, and the proposed method of using contrast sensitivity as measure for partial vision loss should be further explored.

Comments on revisions:

The authors have thoroughly addressed all points raised in my original review, and I have no further concerns.

# Author comment: Author response

**DOI:** 10.7554/eLife.105930.3.sa4

**Authors:** Hugo T Chow-Wing-Bom, Matteo Lisi, Noah C Benson, Freya Lygo-Frett, Patrick Yu-Wai-Man, Frederic Dick, Roni O Maimon-Mor, Tessa Dekker

**Affiliations:** Institute of Ophthalmology, University College London (UCL) London United Kingdom; Royal Holloway University of London London United Kingdom; University of Washington Seattle United States; University College London London United Kingdom; University of Cambridge Cambridge United Kingdom; Birkbeck University of London London United Kingdom; University College London London United Kingdom; University College London London United Kingdom

The following is the authors’ response to the original reviews

**Reviewer #1 (Public review):**

The current claims should be better supported by more evidence.

R1-1: In the first experiment, have the statistics undergone multiple comparison corrections (e.g., Line 441-442)? Given the small sample size, incorporating additional statistical tests (such as the Bayes Factor) could strengthen the analysis.

We confirm that corrections for multiple comparisons are now applied where appropriate, particularly in the group-level ANOVA analyses.

“Post-hoc tests using Holm-Bonferroni correction show that V1 neuronal populations receiving inputs from the central visual field (0.5-4.5°) showed greater contrast sensitivity to high spatial frequency as compared to low spatial frequency stimuli (steeper slope for the 3cpd versus 0.3cpd condition: 0.5-2.5º: *t*(6) = 4.35, *p_bonf_* = 0.0149; 2.5-4.5º: *t*(6) = 3.471, *p_bonf_* = 0.0266). Conversely, peripheral eccentricities in V1 (above 9.5°) showed higher contrast sensitivity to low as compared to high spatial frequency stimuli (steeper slope for 0.3cpd versus 3cpd condition: 9.5-15º: *t*(6) = −4.591, *p_bonf_* = 0.0149; 15-20º: *t*(6) = −6.615, *p_bonf_* = 0.0029). Between 4.5° and 9.5°, V1 contrast sensitivity was similar for both spatial frequencies (*t*(6) = −0.226, *p_bonf_* = 0.8286). Crucially, these effects remained when using retinotopic estimates based on structural scans derived from the Benson retinotopic atlas instead of the pRF-mapping measures (0.5-2.5º: 𝑡(6) = 5.768, *p_bonf_* = 0.0059 ; 2.5-4.5º: *t*(6) = 2.531, *p_bonf_* = 0.0892 ; 4.5-9.5º: 𝑡(6) = −0.293, *p_bonf_* = 0.7792; 9.5-15º: *t*(6) = −3.274, *p_bonf_* = 0.0509; 15-20º: *t*(6) = −3.528, *p_bonf_* = 0.0496; see Figure A2 and Table A3 in Appendix section).”

“Post-hoc pairwise comparisons using Holm-Bonferroni corrections revealed that, as predicted, the cortical contrast response function had a higher slope – indicating better V1 sensitivity – along the horizontal versus vertical quadrants (Horizontal-Vertical Anisotropy – HVA: 𝑡(6) = 5.908, *p_bonf_* = 0.0031) and along the lower versus upper quadrant (Vertical Meridian Anisotropy – VMA: 𝑡(6) = 4.106, *p_bonf_* = 0.0126). Conversely, no difference in cortical contrast sensitivity was found between V1 neuronal populations encoding the left and right quadrants of the visual field (Left-Right Horizontal Meridian Anisotropy – LRHMA: *t*(6) = 0.7197, *p_bonf_* = 0.4988).”

“We found that the horizontal-vertical anisotropy effect was recovered (HVA: *t*(6) = 3.584, *p_bonf_* = 0.0347), but that the vertical meridian anisotropy effect was not (VMA: *t*(6) = 0.744, *p_bonf_* = 0.9697) with this approach.”

R1-2a: The authors claim that "structure-based atlases can replace the need for pRF mapping in cases where it might otherwise be difficult or impossible to collect pRF data." This claim needs further scrutiny. Currently, only one simulated condition of visual field loss was examined in one subject.

AR-R1-2a: We agree that further work is needed to fully establish the utility of structure-based atlases. As a first step, we have followed the reviewer’s suggestion and collected an additional dataset from one of the seven participants, in whom we simulated another condition of visual field loss – specifically, loss of the upper right quadrant. This participant is the same individual already presented in the manuscript (C5), but with a different simulated vision loss condition.

This new condition has been introduced in the Methods, Results and Discussion section, and a new Figure 10 alongside Figure 9 which showed the 3º-8º scotoma. With relevant changes as follows:

“We also demonstrate the clinical relevance of this approach by recovering simulated scotomas (i.e., a ring of visual field loss around fixation and the loss of an entire visual field quadrant), as well as visual field loss in a patient with a neurodegenerative disorder causing large areas of visual field loss.”

“Additionally, one participant (C5) repeated the task under two simulated vision loss conditions (ring or quadrant loss), and two others (C5, C6) completed it with different levels of eye movement.”

“Simulated vision loss

One healthy control participant (C5) also performed a version of the task designed to simulate two forms of visual input loss (i.e., artificial scotoma). These simulations were implemented by: (a) masking a region of the visual field with a grey, annular ring, covering 3º-8º eccentricity, and (b) masking the upper right visual quadrant using a grey quarter-sector overlay. The stimuli and contrast levels used in this task were identical to those described in the original task.”

“A test-case of simulated loss of visual inputs

In the previous sections, we showed that the slope of a square root function provides a reliable measure of contrast sensitivity in the brain of healthy controls. But can this brain-level model also quantify loss of visual inputs? To test this, we first simulated an artificial scotoma in one normal sighted participant, by (a) masking a region of the visual field with a grey, annular ring, covering 3°-8° eccentricity (Figure 9A), and (b) masking the upper-right visual quadrant using a grey quarter-sector overlay (Figure 10A). We expect smaller slope values in V1 neuronal populations that would under normal circumstances encode that part of the visual space.

As expected, we observed reduced responses in V1 locations corresponding to the artificial scotoma (Figures 9 and 10), with increased responses along the edges of the mask for the ring scotoma condition (Figure 9B). This artificial loss of visual input was also clearly present in the cortical contrast sensitivity estimate, with significantly reduced slope steepness in V1 between 3-8° for the ring scotoma condition (Figure 9C&D) and in the upper-right quadrant for the quarter-sector scotoma condition (Figure 10B&C). Additionally, we could recover this scotoma using the calibrated Benson template, although less accurately (Figures 9E and 10D). These results show that this measure of V1 contrast sensitivity is sensitive enough to detect loss of visual inputs in the brain at an individual level, when a complete local loss of sight is simulated, and that this approach does not crucially rely on pRF mapping data from the individual. This supports the utility of our approach in recovering patterns of vision loss and recovery at a cortical level.”

“Mapping Simulated and Pathology-Driven Vision Loss

Our method successfully identified both simulated retinal loss in a healthy volunteer and real visual field loss in a patient with Leber Hereditary Optic Neuropathy (LHON). The signal drop observed in response to masking portions of the visual field in the healthy control was both large and significant at the individual level, as demonstrated by non-overlapping 95% confidence intervals (Figures 9B-C and 10B). This provides proof-of-concept evidence that our approach can detect signal changes in individual patients, which is a critical requirement for clinical translation.

Unlike previous fMRI studies that used high-contrast stimuli (Farahbakhsh et al., 2022; Pawloff et al., 2023; Ritter et al., 2019), which may not accurately represent partial vision loss due to potential saturation effects and the stimulation of less sensitive retinal cells, our use of multiple contrast levels offers a more nuanced assessment of cortical contrast sensitivity.

Combined with the large-field set-up allowing stimulation up to 20° eccentricity, this approach may be particularly well-suited for evaluating treatment efficacy in cases of widespread and variable vision loss.

Future work will focus on further validating reconstruction accuracy under controlled conditions, including simulated scotomas of varying severity and location, expanding testing to larger patient cohorts, and establishing a normative dataset to contextualize patient data.

R1-2b: Also, in Figure 7, contrast sensitivity in the periphery differs between pRF mapping and the Benson atlas. How do the authors explain this discrepancy?

AR-R1-2b: The discrepancy in periphery between pRF mapping and Benson atlas is caused by various factors. These include (a) individual differences in the retinotopy/structure relationship that are not captured in the template, (b) the fact that the Benson atlas at larger eccentricities was obtained with hemifield stimulation, and (c) a larger impact of any inaccuracies at larger eccentricities because of cortical magnification. As a result, peripheral vertices are more likely to be mis-assigned by the template than central ones. Note that this adds distortion in cortical visual field maps which will be consistent across timepoints (rather than noise). Critically, a reduction in accuracy does not preclude utility if meaningful differences in spatial patterns in cortical sensitivity can still be recovered, as is the case in our data. We cover this in the discussion.

“Particularly at large eccentricities however, we initially observed inaccuracies between the template and individual retinotopy eccentricity estimates which led to substantial distortions in cortical visual field maps due to cortical magnification (see Figure A4 in Appendix section). To address this, we adjusted the Benson eccentricity estimates to align with the cortical magnification scaling function (Horton & Hoyt, 1991).”

“Beyond ROI considerations, we still observed differences in cortical sensitivity between pRF mapping and the adjusted Benson atlas - particularly in the periphery. Several factors likely contribute to this. First, individual differences in the relationship between cortical structure and retinotopy are not fully captured by the template. Second, the Benson atlas has never been fit with empirical data more eccentric than approximately 20°, which naturally limits its precision in the far periphery. Third, because of cortical magnification, any small inaccuracy at larger eccentricities has a disproportionately large effect, making peripheral vertices more susceptible to mis-assignment than central ones. These influences introduce systematic distortions in cortical visual field maps rather than random noise and thus remain consistent across time points - an important point when assessing longitudinal changes (e.g., ageing or gene-therapy interventions). Importantly, the spatial gradients in cortical contrast sensitivity were preserved across both the pRF and Benson atlas approaches, indicating that minor ROI differences do not affect our conclusions. Together, these findings show that the Benson Atlas remains a useful alternative when pRF mapping is not feasible.

R1-3: Overall, the writing could be significantly improved.

AR-R1-3: We have made edits throughout the manuscript and hope this has improved the writing.

**Reviewer #1 (Recommendations for the authors):**

R1-Recommendation 1a: The writing can be significantly improved for clarity.

The introduction section is not well-organized, and the motivation for developing the current method (Paragraphs 2-3) is vague and lacks adequate documentation.

Several references are missing (e.g., Lines 90-92) or incorrectly placed (e.g., Lines 108-109).

AR-R1-Recommendation 1a: We have revised the Introduction to clarify the motivation for developing the current method and to correct missing or misplaced references.

“Still, testing visual function across the visual field remains limited in clinical and therapeutic contexts, especially in patients with drastic central vision loss. In this study, we aimed to address this gap by introducing a novel fMRI-based approach to measure visual field sensitivity across a wide expanse of the visual field (40º diameter).”

“Beyond visual acuity, functional impairment across the wider visual field can be measured using a range of visual field tests, from the finger counting visual confrontation field test to more complicated and/or computerized tests (e.g., standard automatic perimetry, kinetic perimetry, microperimetry; Rai et al., 2024). Computerized tests typically involve measuring sensitivity to the luminance contrast of a target relative to a background at different visual field locations while the participant’s gaze is fixed on a central point. In some cases (e.g., microperimetry), sensitivity measurements are paired with fundus imaging, offering greater precision in linking visual field functions to specific retinal locations (Rai et al., 2024). As a result, visual field assessments can reveal functionally relevant deficits – including localized sensitivity loss and scotomas – that are not captured by foveal acuity alone, and are therefore potentially valuable for tracking disease progression and therapeutic efficacy.

Despite their clinical relevance, visual field testing comes with challenges and limitations, and as a result, the inclusion of visual field measures in sight-rescuing therapy trials is limited. Firstly, it requires prolonged fixation and sustained visual attention. This can be very challenging for patients with severe vision loss, who often struggle to fixate, and strain to detect even high intensity stimuli. This can lead to long and unpleasant testing sessions with unreliable results. Secondly, as perception of light stimuli is inherently subjective (Rai et al., 2024) and effortful, patients may vary in their criteria for visual recognition, and in their ability to report visual signals that are weakened or distorted by disease. Together, these constraints reduce the feasibility, robustness, and interpretability of conventional visual field testing in clinical trials, underscoring the need for alternative or complementary approaches that can assess functional vision while placing fewer demands on subjective reporting.”

“Functional MRI (fMRI) has recently been proposed as a promising alternative to measure visual field loss, as it requires no overt task, and instead measures visual sensitivity directly from brain responses (Farahbakhsh et al., 2022; Prabhakaran et al., 2021; Ritter et al., 2019). Population receptive field (pRF) mapping fMRI can measure which parts of the cortex respond to which parts of the visual scene (Dumoulin & Wandell, 2008).”

“Finally, most studies use a single maximum contrast stimulus to assess visual function (Broderick et al., 2022; Farahbakhsh et al., 2022; Liu et al., 2006; O’Connell et al., 2016; Ritter et al., 2019).”

R1-Recommendation 1b: The strengths of the current method and its applicable scenarios are unclear. For example, in Lines 39-40: "We developed an fMRIbased approach to measure contrast sensitivity across the visual field without the need for precise fixation." To what extent can fixation be imprecise? Could this protocol be applied to patients with strabismus, who have biased fixation?

AR-R1-Recommendation 1b: We agree with the reviewer that the tolerance to fixation challenges is key here and so we collected additional data to respond to your points regarding the effects of eye movement on the cortical contrast sensitivity maps.

In terms of biased fixation, the approach should be very robust to this, as this would just reduce the cortical visual field covered on one side and extend it on the other.

We collected new data to test the tolerance to fixation instability across a wide range of eye movement, including severe nystagmus-level movement. Despite large eye movements, the cortical contrast-sensitivity pattern remained largely consistent, though extreme movements reduced slope estimates and flattened the cortical sensitivity pattern for 3cpd, indicating reduced measurement sensitivity for extreme eye movement to high spatial frequency gratings.

These additions have been incorporated into the Abstract, Methods, Results, and Discussion sections as follows:

Abstract

“To assess the method’s tolerance to fixation variability, we further investigated how different levels of eye movement affect cortical sensitivity patterns in two participants. We found that cortical sensitivity patterns were largely preserved across eye movement, particularly at low spatial frequencies. This suggests that our approach can accommodate several degrees of fixation instability, making it suitable for populations with unstable or biased fixation for whom visual field maps are harder to acquire behaviorally (e.g., patients with dense central scotoma or strabismus).”

Methods

“Additionally, one participant (C5) repeated the task under two simulated vision loss conditions (ring or quadrant loss), and two others (C5, C6) completed it with different levels of eye movement.”

Results

“Effect of eye movement

Participants C5 and C6 also performed a version of the task designed to test the effect of eye movements. In this version, saccades were elicited by randomly and rapidly shifting the fixation dot away from central fixation (C5: 2º and 5º from fixation and random motion; C6: up to 2º from fixation). Participant C5 was tested using 0.3 and 3cpd gratings at four contrast levels (7.5, 42.2, 60, 100%), while participant C6 was tested only under the low spatial frequency condition (0.3cpd).

Fixation stability was assessed for each fMRI run using the bivariate contour ellipse area (BCEA), which estimates the area (in degrees^2^ or arcmin^2^) of an ellipse that contains approximately 95% of fixation points. BCEA was calculated using the formula: , as described by Morales et al. (2016). In this expression, *σ_h_* and *σ_v_* represent the standard deviations of eye position in the horizontal and vertical directions, respectively, while *p* corresponds to the Pearson correlation coefficient between horizontal and vertical eye positions. The constant *k* determines the size of the ellipse based on the desired probability area, defined by the relationship *P* = 1 – *e^-k^*, with P set to 0.95 in this study. A smaller BCEA indicates greater fixation stability.

“Effect of eye movements on V1 cortical sensitivity

So far, we have demonstrated that our measure of cortical sensitivity can reliably recover known gradients in sensitivity across eccentricities and visual quadrants. We also showed that this measure was consistent across visits and sessions, suggesting its potential utility for monitoring changes over time. However, all prior tasks were conducted under conditions of central fixation, with participants instructed to maintain gaze on a central dot. A key motivation for this approach was its theoretical robustness to fixation instability. We therefore also aimed to investigate how varying degrees of eye movement might influence cortical sensitivity across the visual field.

To address this, two participants (C5 and C6) completed a modified version of the contrast sensitivity task in which they made eye movements either by following a dot moving randomly at a radius of 2º or 5º around fixation, or by self-initiated very large eye movements. Eye movements across these or by self-initiated very large eye movements. Eye movements across these conditions (Figure 7, bottom row; Figure 8, bottom row), were quantified using BCEA (C5 – Central fixation: mean± SD = 0.57±0.11 deg^2^, 2º eye motion: 2.69±0.48 deg^2^, 5º eye motion: 20.3±1.32 deg^2^, random eye motion: 133.7±23.36 deg^2^; C6 – Central fixation: 0.96±0.56 deg^2^, 2º eye motion: 1.28±0.15 deg^2^). For reference, in severe (idiopathic) nystagmus, the eye movement variability along the vertical and horizontal planes is on average 1.08 deg and 1.60 deg, respectively (Tailor et al., 2021). Assuming a moderate correlation between axes (*p* = 0.3), the average fixation stability would equate to a BCEA of ~21.46 deg^2^ (i.e., ~5º eye motion condition in our data).

Despite these very large levels of eye movements, we observed that the overall cortical contrast sensitivity spatial pattern across eccentricity remained remarkably consistent (Figure 7, top and middle rows; Figure 8, top row). However, at the most extreme movements, contrast sensitivity estimates (slope values) were lower; and while the overall cortical visual field map structure was still clearly present for low spatial frequencies, it appeared more flattened for 3cpd, suggesting reduced sensitivity of our measure for large eye movement and high spatial frequency stimuli.”

Discussion

“Crucially, one advantage of cortical visual field mapping is that the maps are inherently centered on the foveal confluence, providing a stable reference point for comparing responses across eccentricities. When combined with large-field, spatially homogeneous stimuli, this anchoring means that our approach should remain robust to moderate fixation variability and still quantify sensitivity changes across the visual field – provided that fixation instability does not exceed the stimulus extent (40º diameter).

When measuring the impact of eye movements, we found that spatial sensitivity patterns were largely preserved, even for extreme eye movements (emulating severe nystagmus). However, under the most extreme conditions, sensitivity estimates (i.e., slope values) were reduced, especially for high spatial frequency (SF) stimuli. This likely reflects image blurring from large rapid eye movements, which degrades high-SF inputs and shifts activation toward neurons tuned to lower SFs. This aligns with evidence that nystagmus and large saccades impair perception of fine detail and grating stimuli due to retinal image slip (Abadi & Bjerre, 2002; Dickinson & Abadi, 1985; Hertle et al., 2017; Randall et al., 2020). While classic findings report suppression of low-SF signals during saccades (Burr et al., 1994; Ross et al., 2001), our results suggest that high SF sensitivity may be more vulnerable to large eye movements when participants are presented with 2Hz phase-flickering gratings. Further validation in clinical groups with naturally-occurring fixation instability would further strengthen these conclusions.”

R1-Recommendation 1c: There are also some confusing descriptions, such as Lines 130-132.

AR-R1-Recommendation 1c: We have also clarified ambiguous descriptions of the Benson atlas templates.

“We therefore also evaluated the approach using the structure-based atlas of retinotopic values developed by Benson et al. (Benson et al., 2014; Benson & Winawer, 2018). This atlas predicts retinotopic organization by aligning individual cortical anatomy (e.g., surface curvature) to a group-average template that incorporates an algebraic model of retinotopy (Benson et al., 2014). Once the subject’s brain is aligned to this structural atlas, retinotopic maps defined by the model – i.e., polar angle and eccentricity maps – are projected onto the individual’s cortex. This allows estimation of visual field maps without requiring functional imaging, and provides a non-invasive, anatomy-driven approximation of visual field representations.”

R1-Recommendation 1d: Line 361: "Assessing the brain's ability to discriminate shapes"-is the author referring to the functional relevance of contrast tuning assessment here? Since the task or stimuli are not related to shapes, this description is unclear.

AR-R1-Recommendation 1d: We have revised the reference to “discriminating shapes” to more accurately reflect the functional relevance of contrast sensitivity mapping.

“To measure visual field function, we developed a new measure of cortical contrast sensitivity, assessing the brain’s ability to discriminate gratings of varying spatial frequencies based on luminance variations.”

R1-Recommendation 2a: Simulated visual loss experiment: only one condition of visual field loss was examined in a single subject. I encourage the authors to include additional subjects to meet statistical test criteria at group level. Simulated scotomas in more visual quadrants, including both central and peripheral areas, should be examined, as asymmetries may exist.

AR-R1-Recommendation 2a: We agree that it is important to verify that the approach can also capture other types of scotomas. We have therefore now incorporated another simulated condition of visual field loss, namely loss of the upper right quadrant.

Regarding adding more participants: The drop in signal is clearly large and significant at the individual level (error bars corresponding to 95% confidence interval do not overlap; Figures 9B-C & 10B). The ability to detect signal change at the individual level is what we need for clinical application, and here we are showing proof-of-concept of its feasibility with our approach. However, we do appreciate that it might be valuable to test cortical visual field loss reconstruction accuracy with simulated scotomas of varying levels of vision loss in variable locations. We now highlight this as a future direction.

Please refer to our response to R1-2a, where we also detail the corresponding changes made in the manuscript.

R1-Recommendation 2b: Additionally, why do the results from pRF mapping and the corrected Benson atlas differ, particularly in the far periphery?

AR-R1-Recommendation 2b: Please refer to our response to R1-2b, where we also detail the corresponding changes made in the manuscript.

R1-Recommendation 3: To validate the recovery of visual field loss in the case study, it would be necessary to include fundus imaging to characterize the structural loss and correlate it with the behavioral and fMRI results.

AR-R1-Recommendation 3: We included Compass perimetry data for the LHON patient, which is fundus-tracked perimetry and uses fundus imaging to keep the visual stimulation fixed to retinal locations.

In the context of LHON, the fundus image is not expected to provide more information than perimetry. This is because the visual deficit in LHON arises from optic nerve dysfunction, and retinal abnormalities are typically minimal. Aside from the characteristic pallor of the optic disc, the fundus appearance is usually normal in appearance.

For illustration, Author response image 1 shows the Compass-acquired fundus image from the LHON patient included in this study. For comparison, we also show a normal fundus image from a 25-year-old male volunteer, reproduced from Häggström, Mikael (2014). "Medical gallery of Mikael Häggström 2014". WikiJournal of Medicine 1 (2). DOI:10.15347/wjm/2014.008. ISSN 2002-4436. Public Domain.

**Author response image 1.** 

We do, however, recognize the importance of linking functional changes to structural alterations (e.g., retinal thickness measured with OCT), and we now highlight this as a key future direction in the discussion. This will be a central focus of a planned follow-up study involving a larger patient cohort.

“Next steps in this work will therefore involve testing larger patient cohorts with diverse forms of vision loss, validating the approach for tracking pathology over time, and investigating how cortex-based visual field measures relate to and complement other visual field and retinal integrity indices including Compass measures and OCT-derived retinal layer thickness.”

“Additionally, linking brain-based variations in function across the visual field to behavioral performance (e.g., perimetry, microperimetry) and retinal structure (fundus imaging, retinal thickness from Optical Coherence Tomography), could help bridge the gap between neural measures and functional outcomes. Such integration would provide deeper insights into developmental, learning, and vision loss mechanisms.”

R1-Recommendation 4a: Why is a 0.5 mm smoothing applied to the contrast task data?

AR-R1-Recommendation 4a: We have now clarified in the Methods section. This 0.5 mm FWHM smoothing kernel was applied to the contrast sensitivity task data to meet the minimum requirements of the GLM module in SPM.

“To accurately capture neural activity across various eccentricities and polar angle locations, minimal smoothing (0.5mm FWHM Gaussian blur) was applied to the contrast sensitivity task data using FSL’s 3dmerge program. This was done to meet the minimum requirements of the GLM module in SPM.”

R1-Recommendation 4b: Is this the first time the cortical magnification calibration has been applied to the Benson atlas? I recommend including a figure to describe this method.

AR-R1-Recommendationn 4b: This is indeed the first time this correction has been applied to the Benson atlas. We have now added a figure (Figure 3) to illustrate the eccentricity adjustment procedure applied to the Benson atlas.

R1-Recommendation 5: In Figure 5, the test-retest reliability can be reported by including r-values.

AR-R1-Recommendation 5: We have now included Spearman correlation 𝜌-coefficients for test-retest and between-condition comparisons in Figure 6 (previously Figure 5).

R1-Recommendation 6: Inconsistency in the reporting format of statistical values: e.g., the degrees of freedom are presented with, or without parentheses.

AR-R1-Recommendation 6: Thank you for pointing this out. We have reviewed and standardized the reporting format of all statistical values throughout the manuscript to ensure consistency. Degrees of freedom are now all presented with parentheses, in details:

“Using ANOVA, we found the expected interaction between spatial frequency and eccentricity (*F*(1.96,11.79) = 28.66, p < 0.001; Figure 4) as well as a main effect of eccentricity (*F*(2.33,13.99) = 12.67, *p* < 0.001).”

“We found a main effect of visual field quadrant location on V1 sensitivity (*F*(2.46,14.76) = 20.71, *p* < 0.001).”

“Moreover, there was no interaction between spatial frequency and (*F*(2.16,12.99) = 1.34, *p* = 0.298), visual field quadrant positions suggesting V1 visual field anisotropies are relatively constant across spatial frequencies.”

**Reviewer #2 (Public reviews):**

R2-1a: Questionable sensitivity to differences in patients. The variability in heat maps across healthy control participants is somewhat surprising. Do differences between individuals represent actual visual sensitivity differences, or are they an artifact of the measurement technique, e.g., due to signal-to-noise differences introduced by local variations in brain anatomy? Will the substantial variance across controls allow for a sufficiently stable baseline to detect meaningful differences in individual patients?

AR-R2-1a: We agree the variability across healthy controls is surprising. It is unclear whether this reflects true individual differences in visual sensitivity or arises from factors like local signal-to-noise introduced by local variations in brain anatomy. It will be really interesting to investigate this further by examining structural variations across the visual field and comparing them with behavioral measures.

As for establishing a stable baseline for patient comparisons, this is inherently an empirical question and depends on the degree of vision loss. LHON patients typically show dense central scotomas (up to 15º) in the chronic phase, making them well suited for detecting sensitivity differences – e.g., between central versus peripheral locations. Detecting subtler changes – in the acute phase or other conditions – may be more challenging. We agree with the reviewer that a normative range will be essential for contextualizing patient data, which we now mention in the Discussion, and we aim to develop in the future based on the present data.

“Future work will focus on further validating reconstruction accuracy under controlled conditions, including simulated scotomas of varying severity and location, expanding testing to larger patient cohorts, and establishing a normative dataset to contextualize patient data.”

R2-1b: Also, as the authors rightly point out, Benson atlas does not model differences along meridians, so upper/lower field differences might not be detectable.

AR-R2-1b: We acknowledge the limitations of the Benson atlas, particularly its inability to model meridional asymmetries (e.g., upper vs. lower visual field). Still, our goal is to provide a method for tracking visual cortex changes over time. By consistently projecting longitudinal functional data onto the same structural image fitted with the Benson atlas, we maintain a stable anatomical reference, which supports reliable comparisons across timepoints – even with limited spatial accuracy. Future improvements could include shearing corrections, Bayesian updating, or alternative models such as DeepRetinotopy developed by Ribeiro et al.

“Further enhancing the alignment between retinotopic template atlases and individual retinotopic tuning could improve this approach further, for example, by integrating them with functional measures using Bayesian methods (Benson & Winawer, 2018). In parallel, geometric deep learning frameworks such as DeepRetinotopy (Ribeiro et al., 2021) could also offer anatomy-driven predictions from structural MRI, and combining these strategies may yield more accurate and generalizable retinotopic reconstructions.”

R2-2: Effects of unstable fixation/eye movements not explicitly tested: The methods state, 'In all tasks, participants were asked to report when the color of a central fixation dot changed', suggesting participants maintained fairly good fixation. Most of the results seem to pertain to measurements where central fixation is required. How does unstable fixation affect measurements?

AR-R2-2: This is an important point. We have now extensively and systematically investigated the impact of eye movements on the cortical contrast sensitivity maps and updated the Abstract, Methods, Results, and Discussion sections (see R1-1b).

R2-3: Potential for clinical translation. Although it is a sensitive measure, functional MRI is costly, is not available in all clinical settings, requires significant post-processing analyses, and may be contraindicated in some individuals due to safety (e.g., metallic implants) or other concerns (e.g., claustrophobia). These could present significant barriers to widespread clinical translation if this were the ultimate goal of the study.

AR-R2-3: We agree that fMRI, while sensitive, has practical limitations for broad clinical adoption due to cost, accessibility, and contraindications. However, it remains a valuable tool in targeted contexts, where sensitive detection of visual field loss has large utility – for example for evaluating treatment effects in clinical trials. This application has been demonstrated in recent studies (Farahbakhsh et al., 2022; Maimon-Mor et al., 2025; Haal et al., 2016; Ritter et al., 2019).

R2-4: Limited range of spatial frequencies. The spatial frequencies tested were still quite low (0.3 and 3cpd) compared to measures such as visual acuity. Extending the measurements to higher spatial frequencies could allow better characterization of central vision, although necessarily for peripheral vision.

AR-R2-4: We agree that extending to higher spatial frequencies could improve central vision characterization and note this can be readily incorporated into future studies using the current framework. However, LHON patient’s acuity tends to be very low, and we found that 5cpd did not allow us to measure any cortical contrast sensitivity in a prior pilot. So, to characterize the visual field in LHON with fMRI, we therefore aimed to balance central and peripheral coverage: 0.3 cpd ensured broad detectability, while 3 cpd offered a middle ground to assess central vision without exceeding acuity of this population. Additional approaches, such as neural contrast sensitivity functions (e.g., Roelofzen et al., 2025) may also offer complementary insights such as acuity, and contrast sensitivity across the full spatial frequency range (area under the curve).

**Reviewer #2 (Recommendations for the authors):**

R2-Recommendation 1: It appears that the reliability measures, comparing differences in Spearman correlations between and within sessions, were not tested statistically, but evaluated qualitatively. What was the justification for this? The results only state Spearman values, but the discussion claims that the differences between the two comparisons were significant.

AR-R2-Recommendation 1: The differences in Spearman correlations between and within sessions were tested statistically, and the omission of p-values was an oversight. We have now revised the Results section results from the paired one-tail t-test as follows:

“We collected test-retest reliability measures from 4 out of 7 participants (Figures 6A-B) and benchmarked them against the correlations between the 0.3cpd condition and 3cpd spatial frequency condition, collected in the same session (Figure 6C). If measures are reliable, correlations should be higher for repeated measures with the same spatial frequency stimulus, collected on different days. We tested this prediction using a one-tailed paired t-test.”

“This difference was statistically significant (*t*(3) = 2.62, *p* < 0.0395).”

R2-Recommendation 2a: The variability of heat maps (visual field sensitivities) between healthy controls should also be discussed. What are potential explanations for this variability?

AR-R2-Recommendation 2: We have expanded the Discussion section to address the variability observed in cortical sensitivity maps across healthy controls.

“We also observed intriguing variability in cortical visual field maps across healthy controls, and this variability was consistent across measures. This may reflect genuine individual differences in visual sensitivity that are relevant for behavioral performance. Alternatively, it could arise from factors such as local signal-to-noise differences driven by anatomical variability. However, the fact that maps derived from different spatial stimulus conditions showed markedly different patterns argues against a purely anatomical explanation and suggests that at least part of the variability is functional. Despite this inter-subject variability, variations in cortical contrast sensitivity across eccentricities and visual field quadrants were significant at the individual level indicating high sensitivity.”

R2-Recommendationn 2b: There should also be more discussion about any potential effects of eye movements/unstable fixation in order to address the suitability of the methods for these clinical populations.

AR-R2-Recommendation 2b: Please refer to our response to R2-2, where we also detail the corresponding changes made in the manuscript.

**Reviewer #3 (Public review):**

R3-1: The authors should more strongly emphasize their findings on the organization of contrast sensitivity, particularly in light of the stimulation extent provided by the wide-field setup.

AR-R3-1: Thank you for this important point – we have now emphasized more clearly in the manuscript that our method extends the measurement of contrast sensitivity to 20º eccentricity, which represents a significant advancement over previous studies.

“These results demonstrate that our approach can detect subtle changes in visual sensitivity across eccentricities at the individual participant level. The ability to reveal these gradients was made possible by the large peripheral coverage provided by our large-field stimulation set-up (see Figure A1 in Appendix section), which enabled a more complete characterization of V1 sensitivity across the visual field. Importantly, the same effects were preserved when using retinotopic estimates derived from structure-based atlases, demonstrating that atlas-based methods can be used as alternative to pRF mapping in cases where it might otherwise be difficult or impossible to directly collect pRF measures. Together, these highlight both the validity of our approach and its potential to broaden the scope of visual neuroscience.”

“Crucially, the ability to visualize these sensitivity gradients was made possible by the large peripheral coverage provided by our large-field stimulation set-up. Such coverage is particularly important for clinical applications, as it enables the detection of visual field losses beyond the macula (i.e., beyond 10º eccentricity) and the evaluation of residual peripheral vision in patients with macular-restricted damage. In doing so, this work provides a useful tool for advancing both basic visual neuroscience and translational research in clinical populations.”

R3-2: Certain methodological aspects require further clarification, particularly regarding the correction of eccentricity values from the Benson atlas. It's not clear which V1 masks are used for the specific analysis which could have a substantial impact on the reported differences between the two approaches of pRF mapping and atlas-based pRF parameters.

AR-R3-2: The correction of eccentricity values was performed using the V1 label provided by the Benson atlas. We have now explicitly stated this in the Methods section:

“We collected data from 7 healthy controls (mean± SD: 29.6±4.7yo; 1M). All controls either had normal or corrected to normal vision, with no other ocular pathologies, and were recruited from the local staff and student pool at the University College of London. Each control completed both the population receptive field (pRF) mapping and the fMRI contrast sensitivity task. To assess measurement repeatability, four participants (C2, C4, C5, C6) performed the contrast sensitivity task twice. Additionally, one participant (C5) repeated the task under two simulated vision loss conditions (ring or quadrant loss), and two others (C5, C6) completed it with different levels of eye movement.”

“Four participants (C2, C4, C5, C6) were invited for a second session in which they repeated the task to assess the reliability of the measures.”

R3-4: The conclusion that high-contrast patterns as in pRF mapping are not optimal to test for subtle but potentially clinically relevant changes in the visual field coverage is very valid. The suggested use of contrast sensitivity can therefore be a potentially well-suited parameter for estimating visual field losses. The presented work is an interesting starting point and the proposed method of using contrast sensitivity as a measure for partial vision loss should further be explored.

AR-R3-4: Thank you for the positive evaluation of our work.

**Reviewer #3 (Recommendations for the authors):**

R3-Recommendation 1: The shown organization of contrast sensitivities is consistent with previous studies; however, it extends the measurements to up to 20º eccentricity, which is, to my knowledge, much more than previously reported. The authors should therefore emphasize this more strongly.

AR-R3-Recommendation 1: Please refer to our response to R3-1, where we also detail the corresponding changes made in the manuscript.

R3-Recommendation 2: In the Methods section, it is not entirely clear why the eccentricity values originating from the Benson atlas need to be corrected using Horton & Hoyt cortical magnification. Do the authors consider these cortical magnification measurements as ground truth? Is the correction only applied to higher eccentricity values that are not mapped by the Benson atlas?

AR-R3-Recommendation 2: The Benson et al. (2014) atlas predicts both polar angle and eccentricity from cortical anatomy (curvature, thickness) using a template pRF dataset and a mathematical retinotopic model. However, it does not incorporate a smooth parametric cortical magnification function such as Horton & Hoyt. Because the atlas is fit to an average map across subjects, and because the FreeSurfer alignment used to apply the template cannot incorporate functional information, the atlas cannot capture individual variability in eccentricity or cortical magnification. In practice, we therefore treat the Benson atlas as providing the correct topological layout of eccentricity, but not necessarily the correct eccentricity values for a given individual. Moreover, the data used to generate the Benson atlas have mainly been restricted to the central visual field (roughly 8º-12º) and the Benson atlas themselves has never been fit with data more eccentric than 20º. Consequently, peripheral eccentricity values are more model-driven and less constrained by ground-truth data.

To improve the correspondence between the atlas and expected cortical representations, we applied Horton & Hoyt cortical magnification function to all eccentricities in the V1 Benson mask (from the foveal confluence to the periphery, up to 90º). We assume that the Horton & Hoyt model, adapted from physiology data, provides an accurate model of group level cortical magnification (Benson et al., 2021) – even though it does not capture individual differences. This means it offers the best approximation of ground-truth in the absence of individual pRF data, which is often not feasible to collect in patients with unstable fixation. We have now added a figure that showcases the method and shows how this correction affects the distribution of eccentricity values in the Benson atlas.

R3-Recommendation 3: For the analysis using the atlas-based retinotopy, it is not entirely clear whether the authors also use the provided V1 masks. In other words, differences between the original pRF-based and atlas-based analyses could originate from different borders of V1 rather than from the atlas-based pRF parameters. The authors could try using the same mask for both analyses, either the manually delineated one or the atlas-based one.

AR-R3-Recommendation 3: This is a well-noted point that is important to clarify. We used a manually delineated V1 mask for the own pRF map data and the Benson mask for the adjusted Benson atlas-based analysis – both restricted to the screen size. The difference in included vertices could have indeed introduced some additional error beyond the atlas/pRF mapping itself. We have opted not to correct this in this version of the manuscript because (1) the error introduced is likely small (as we inspected that the alignment of V1 ROI delineations with the Benson ROIs are good, so effects are likely not too major - although using identical masks may slightly improve the mapping further in particular the very center and outer-periphery), and (2) our ROI selection for each respective approach is in line with typical procedures used in reality. Critically, the spatial gradients in cortical contrast sensitivity are preserved across the pRF and Benson atlas approach with the different ROIs, so we believe that improvements would not alter our conclusions that Benson offers a useful alternative when pRF mapping is not possible - however, we now highlight this important difference across the two approaches in the paper.

“With this structure-based atlas, we successfully replicated key variations in visual field function (across eccentricity and polar quadrants), although sensitivity to more subtle differences (e.g., upper versus lower quadrant anisotropy) was reduced. This reduction may partly stem from differences in ROI definitions: a manually delineated V1 mask was used for the pRF-based data, while the Benson atlas mask was used for the adjusted Benson atlas analysis. Such differences could introduce minor error beyond the atlas/pRF mapping itself due to differences in the vertices included by each mask.”

“Importantly, the spatial gradients in cortical contrast sensitivity were preserved across both the pRF and Benson atlas approaches, indicating that minor ROI differences do not affect our conclusions. Together, these findings show that the Benson atlas remains a useful alternative when pRF mapping is not feasible.”

R3-Recommendation 4: The patient was measured monocularly. Given the widefield stimulation setup and the fact that the blind spot is located at about 15º eccentricity, do the authors expect to measure this blind spot with the given setup?

Does this have an influence in binocular measurements?

AR-R3-Recommendation 4: This is an interesting point. In theory, our wide-field setup could allow for the detection of the blind spot, as located around 12-15º eccentricity. However, in our LHON patient, the visual field defect typically extends to or beyond the blind spot, making it difficult to isolate its boundary, as shown in Figure 11 (previously Figure 7). Additionally, under binocular viewing, the brain integrates inputs from both eyes to create a unified percept, which may obscure blind spots unless specific paradigms are used (e.g., binocular rivalry or dichoptic tasks). Whilst this is outside the scope of this work, our setup could be adapted to map out the blind spot or explore phenomena like binocular rivalry more directly in future research.

R3-Recommendation 5: How stable is the presented wide-field stimulation setup? In other words, does the eye tracker still capture the eye reliably after small head movements?

AR-R3-Recommendation 5: While small head movements can occur, these were minimized by the use of padding cushions and monitored throughout the session, and the eye tracker maintained reliable tracking throughout the sessions.

R3-Recommendation 6: Are the shown sine-wave gratings always oriented the same? We would expect orientation tuning curves in the early visual cortex; how could this influence the results?

AR-R3-Recommendation 6: For six of the seven control participants (C1-C6), the sinewave gratings were presented with a fixed horizontal orientation. In an updated version of the task – used for participant C7, cases of simulated eye movements, cases of artificial scotoma, and the patient – the orientation of the gratings was varied every 5 seconds among four angles (−45º, 0º, 45º, 90º) during each 15-second stimulus block.

We acknowledge that orientation tuning in the early visual cortex could influence responses, since V1 neurons are selective for specific stimulus orientations and respond most strongly to their preferred orientation. However, we replicated the same overall pattern of results in groups tested with a single orientation and with multiple orientations. Importantly, some participants completed both versions of the task, and the contrast sensitivity patterns remained consistent across conditions. This suggests that the results we report are robust across different orientation-tuned populations for the purposes of this study. A more fine-grained investigation of orientation effects would nevertheless be an interesting direction for future work.

“For six control participants (C1–C6), gratings were initially presented with a fixed horizontal orientation. In an updated version of the task – used for C7, cases of simulated eye movement, cases of artificial scotoma, and the LHON patient – the orientation varied every 5 s among four angles (−45º, 0º, 45º, 90º). Contrast sensitivity patterns were consistent across single and multiple-orientation conditions, including in participants who completed both versions, indicating robustness across orientation-tuned populations.”

R3-Recommendation 7: Are pRF centers also fitted outside the stimulated 20º radius? If yes, were they masked for the analysis?

AR-R3-Recommendation 7: During pRF model fitting, pRF centers were allowed to extend beyond the stimulated visual field, up to approximately 1.5 times the maximum stimulus eccentricity (~30°), to improve model stability near stimulus boundaries. Eccentricity was sampled on a logarithmically spaced grid defined as 2^*^, with 𝑥 ranging from -5 to 0.6 in steps of 0.2, and then scaled by the maximum stimulus eccentricity (20°) to express pRF centers in degrees of visual angle. This spacing approach provided finer sampling near the fovea and progressively coarser sampling at larger eccentricities, consistent with cortical magnification principles. For all subsequent analyses of cortical contrast sensitivity, pRF centers located outside the stimulated 20° eccentricity were explicitly excluded. Likewise, although the Benson atlas provides eccentricity estimates extending well beyond the stimulated range (up to ~90°), only pRF centers within 20° were included to ensure consistency across pRF based and atlas-based analyses.

“During pRF model fitting, pRF centers were allowed to extend beyond the stimulated visual field to improve model stability near stimulus boundaries – up to approximately 1.5 times the maximum stimulus eccentricity (~30°). Eccentricity was sampled on a logarithmically spaced grid defined as 2*, with *x* ranging from −5 to 0.6 in steps of 0.2, and then scaled by the maximum stimulus eccentricity (20°) to express pRF centers in degrees of visual angle. This sampling scheme provided finer resolution near the fovea and progressively coarser sampling at larger eccentricities, consistent with cortical magnification principles.”

“For all subsequent analyses of cortical contrast sensitivity, pRF centers outside the stimulated 20° eccentricity were excluded. Similarly, although the Benson atlas provides eccentricity estimates extending far beyond the stimulated range (up to ~90°), only values within 20° were retained to maintain consistency across pRF-based and atlas-based analyses.”

R3-Recommendation 8: L212: Could the authors please clarify what "scaled across eccentricity to account for cortical magnification" means for the given stimulus?

AR-R3-Recommendation 8: The pRF stimulus was scaled across eccentricity using a logarithmic transformation of retinal radius to approximate cortical magnification. Radial checker boundaries were defined in log eccentricity space *(log(r))*, resulting in an exponential increase in checker size with eccentricity (scaling factor = 3.2; ~1.37× increase per radial step). As a result, the spatial frequency content of the stimulus decreases with eccentricity (i.e., checker size increases), compensating for known changes in V1 spatial frequency preference across the visual field. This eccentricity dependent scaling inherently relies on precise fixation to stimulate the intended retinal locations, which can be difficult for patients with central vision loss and therefore motivates the use of Benson templates.

“This scaling was implemented by applying a logarithmic transformation of retinal radius, such that radial checker boundaries were defined in log eccentricity space *(log(r))*, where *r* denotes to eccentricity relative to the fixation target. This produced an exponential increase in checker size with eccentricity (scaling factor = 3.2; ~1.37 times increase per radial step), resulting in lower spatial frequency content at larger eccentricities – consistent with known variations in V1 spatial frequency tuning. Because this eccentricity dependent scaling assumes precise fixation, it can be challenging for individuals with central vision loss, further motivating the use of Benson atlas templates in such populations.”

R3-Recommendation 9: L213: Three runs were measured per session, were they averaged before analysis or analyzed independently? If analyzed independently, how were the individual results handled?

AR-R3-Recommendation 9: As described in the Methods, data from all three runs were first aligned to an alignment scan that had been co-registered to the MPRAGE image – typically the scan with the fewest outlier voxels, or alternatively, a single-band reference scan in cases of misregistration. The runs were then analyzed as separate regressors in a single design matrix in SPM to account for run-specific variation - following standard recommendations for this software (Author response image 2 shows the SPM design matrix for the GLM). We did not average the runs beforehand due to differences in the order of stimulus presentation across runs. Instead, the GLM modeled each run’s specific presentation sequence to estimate condition-specific beta values, capturing the average contribution of each spatial frequency and contrast level to the BOLD response.

**Author response image 2.**

R3-Recommendation 10: L289: Did the authors check for very small pRF sizes, as SamSrf is prone to fitting many small sizes?

AR-R3-Recommendation 10: We did not apply an explicit filter to remove very small pRF sizes; we excluded only pRFs with σ > 6.

R3-Recommendation 11: L384: p is missing before the value.

AR-R3-Recommendation 11: Thank you for catching this oversight. We have now added the missing p-value in the revised manuscript.

“Post-hoc tests using Holm-Bonferroni correction show that V1 neuronal populations receiving inputs from the central visual field (0.5-4.5°) showed greater contrast sensitivity to high spatial frequency as compared to low spatial frequency stimuli (steeper slope for the 3cpd versus 0.3cpd condition: 0.5-2.5º: *t*(6) = 4.35, *p_bonf_* = 0.0149; 2.5-4.5º: 𝑡(6) = 3.471, *p_bonf_* = 0.0266).”

R3-Recommendation 12: I have a very subjective comment regarding the figures. I do not really like the use of the hot colormap in this setting, as I feel it is hard to interpret high and low values.

AR-R3-Recommendation 12: We appreciate the suggestion, but we have had many heated discussions amongst the authors about this and have moved back forth several times before settling. Hopefully the reviewer will be happy for us to stick with the author’s eventually agreed-on subjective preference although we acknowledge that it is by no means a perfect color scheme.

R3-Recommendation 13: L474: Suddenly, a second session appears in the Results section; please report this in Methods.

AR-R3-Recommendation 13: Please refer to our response to R3-3, where we also detail the corresponding changes made in the manuscript.

R3-Recommendation 14: Figure 5C: are the reported results from the first session of the same subjects?

AR-R3-Recommendation 14: That is correct. The results shown in Figure 6C (previously 5C) reflect correlations between slope estimates obtained from the 0.3 and 3cpd conditions within the same session for each subject. We have updated the panel title to “C. 0.3cpd vs 3cpd (within session)” to clarify this point.

R3-Recommendation 15: For the classic pRF mapping (Figure 6D), the artificial scotoma shows lower contrast sensitivity within the scotoma and increased values outside its borders. In contrast, using the retinotopic template (Figure 6E), the area of increased sensitivity is shifted inside the scotoma. Can the authors please comment on this discrepancy?

Is this shift due to systematic differences between the eccentricity values estimated during the pRF run and those derived from the template?

If such a shift exists, is it induced by the eccentricity correction step performed?

AR-R3-Recommendation 15: The shift inside the scotoma observed in the atlas-based analysis (Figure 9E; previously Figure 6E) compared to the pRF-based analysis (Figure 9D; previously Figure 6D) likely reflects residual inaccuracies in eccentricity estimates from the adjusted Benson atlas. While the Horton & Hoyt correction improves the alignment of eccentricity values, it does not ensure perfect matching with the pRF data. Without the Horton & Hoyt correction, the misalignment and shift of activity in the scotoma region are even more pronounced (see below).

We have added a sentence to the Methods section to justify the applied correction. Furthermore, to illustrate the impact of misalignment and its correction on cortical sensitivity maps, we have included an additional figure in the Appendix section showcasing the effect of applying the correction to improve mapping of the artificial scotoma.

“We initially observed inaccuracies between the template and individual retinotopy eccentricity estimates which led to substantial distortions in cortical visual field maps due to cortical magnification – especially in peripheral locations (see Figure A4 in Appendix section).”

R3-Recommendation 16: L532: The age and mutation type of the patient are already reported in the Methods. In general, many Methods and Discussion statements are embedded within the Results section.

AR-R3-Recommendation 16: We are aware that it is a stylistic choice to remind of method in the results and foreshadow discussion. We chose this approach to support the interpretability of the results for less specialist readers.

R3-Recommendation 17: L636: Did the authors consider other options for estimating pRF parameters based on anatomical features, like Ribeiro et al. (2021; here).

AR-R3-Recommendation 17: We agree that alternative approaches to estimating pRF parameters based on anatomical features, such as the DeepRetinotopy method proposed by Ribeiro et al. (2021), are promising and worth exploring. In this study, we used the Benson atlas as a starting point, along with an adjustment of eccentricity estimates based on cortical magnification. Future work could compare the performance of different retinotopic template fitting approaches, including deep learning-based methods, to further improve anatomical alignment and functional predictions.

“Further enhancing the alignment between retinotopic template atlases and individual retinotopic tuning could improve this approach further, for example, by integrating them with functional measures using Bayesian methods (Benson & Winawer, 2018). In parallel, geometric deep learning frameworks such as DeepRetinotopy (Ribeiro et al., 2021) could also offer anatomy-driven predictions from structural MRI, and combining these strategies may yield more accurate and generalizable retinotopic reconstructions.”

R3-Recommendation 18: Figure A4: This figure brings up a very important point, namely, whether small eye movements reduce the accuracy of pRF and contrast sensitivity estimates. However, these experiments and results are not reported in the manuscript. I would prefer the authors to add all necessary Methods and Results, or at least not leave this Figure unexplained.

AR-R3-Recommendation 18: We thank the reviewer for highlighting the importance of this figure. To address this point, we collected additional data and have revised the manuscript to include a dedicated section on the effects of eye movements, with corresponding updates in the Abstract, Methods, Results, and Discussion.
